# Discovery of a New, Recurrent Enzyme in Bacterial Phosphonate Degradation: (*R*)-1-Hydroxy-2-aminoethylphosphonate Ammonia-lyase

**DOI:** 10.1021/acs.biochem.1c00092

**Published:** 2021-04-08

**Authors:** Erika Zangelmi, Toda Stanković, Marco Malatesta, Domenico Acquotti, Katharina Pallitsch, Alessio Peracchi

**Affiliations:** † Department of Chemistry, Life Sciences and Environmental Sustainability, University of Parma, I-43124 Parma, Italy; ‡ Institute of Organic Chemistry, University of Vienna, Währingerstrasse 38, A-1090 Vienna, Austria; § Centro di Servizi e Misure “Giuseppe Casnati”, University of Parma, I-43124 Parma, Italy

## Abstract

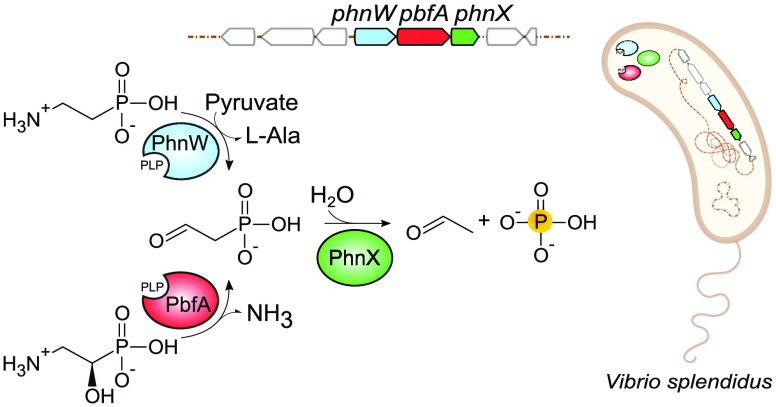

Phosphonates represent an important source of bioavailable phosphorus in certain environments. Accordingly, many microorganisms (particularly marine bacteria) possess catabolic pathways to degrade these molecules. One example is the widespread hydrolytic route for the breakdown of 2-aminoethylphosphonate (AEP, the most common biogenic phosphonate). In this pathway, the aminotransferase PhnW initially converts AEP into phosphonoacetaldehyde (PAA), which is then cleaved by the hydrolase PhnX to yield acetaldehyde and phosphate. This work focuses on a pyridoxal 5′-phosphate-dependent enzyme that is encoded in >13% of the bacterial gene clusters containing the *phnW–phnX* combination. This enzyme (which we termed PbfA) is annotated as a transaminase, but there is no obvious need for an additional transamination reaction in the established AEP degradation pathway. We report here that PbfA from the marine bacterium *Vibrio splendidus* catalyzes an elimination reaction on the naturally occurring compound (*R*)-1-hydroxy-2-aminoethylphosphonate (*R*-HAEP). The reaction releases ammonia and generates PAA, which can be then hydrolyzed by PhnX. In contrast, PbfA is not active toward the *S* enantiomer of HAEP or other HAEP-related compounds such as ethanolamine and d,l-isoserine, indicating a very high substrate specificity. We also show that *R*-HAEP (despite being structurally similar to AEP) is not processed efficiently by the PhnW–PhnX couple in the absence of PbfA. In summary, the reaction catalyzed by PbfA serves to funnel *R*-HAEP into the hydrolytic pathway for AEP degradation, expanding the scope and the usefulness of the pathway itself.

Phosphonate compounds, containing a direct C–P bond instead of the more usual C–O–P ester linkage,^1^ occur in the environment where they constitute an important source of organic phosphorus^2^ and are frequent environmental pollutants.^3^ Phosphonates are difficult to degrade due to their very stable C–P bond, yet several microorganisms possess biochemical pathways that can break down some phosphonates, allowing their utilization as a source of phosphorus.^4,5^ Some of these pathways have been characterized at the biochemical and molecular level and can be schematically distinguished on the basis of the mechanism through which the C–P bond is ultimately cleaved - i.e., through either a hydrolytic, radical, or oxidative reaction.^1,6^

The degradation of phosphonates is particularly advantageous for microbes living in marine environments, where the bioavailability of phosphorus is often a limiting factor for growth.^7^ In particular, many marine bacteria possess a specialized “hydrolytic” pathway for the breakdown of the natural compound 2-aminoethylphosphonate (AEP; also known as ciliatine), which is the most widely distributed biogenic phosphonate in the environment.^4,8^ AEP degradation relies on an initial transamination catalyzed by PhnW, a pyridoxal 5′-phosphate (PLP)-dependent aminotransferase, that converts AEP into phosphonoacetaldehyde (PAA);^9,10^ in turn, PAA is transformed into acetaldehyde and inorganic phosphate by the hydrolase PhnX^11^ (Figure 1A). In a variation of the pathway described above, the PhnW-produced PAA can be first converted to phosphonoacetate and then to acetate and phosphate by the combined action of two other enzymes, termed PhnY and PhnA^12,13^ (Figure 1B). More rarely, AEP is degraded through an oxidative route, where a first oxygenase (called PhnY*) forms the intermediate (*R*)-1-hydroxy-2-aminoethylphosphonate (*R*-HAEP), which is then cleaved by the dioxygenase PhnZ, yielding glycine and phosphate^14−16^ (Figure 1C). Degradation of another common aminophosphonate, l-phosphonoalanine, proceeds again through a hydrolytic mechanism. It begins with the transamination of phosphonoalanine to phosphonopyruvate, operated by the PLP-dependent enzyme PalB; then a metal-dependent hydrolase, PalA, cleaves the phosphonopyruvate C–P bond to yield pyruvate and phosphate^4,17^ (Figure 1D).

**Figure 1:**
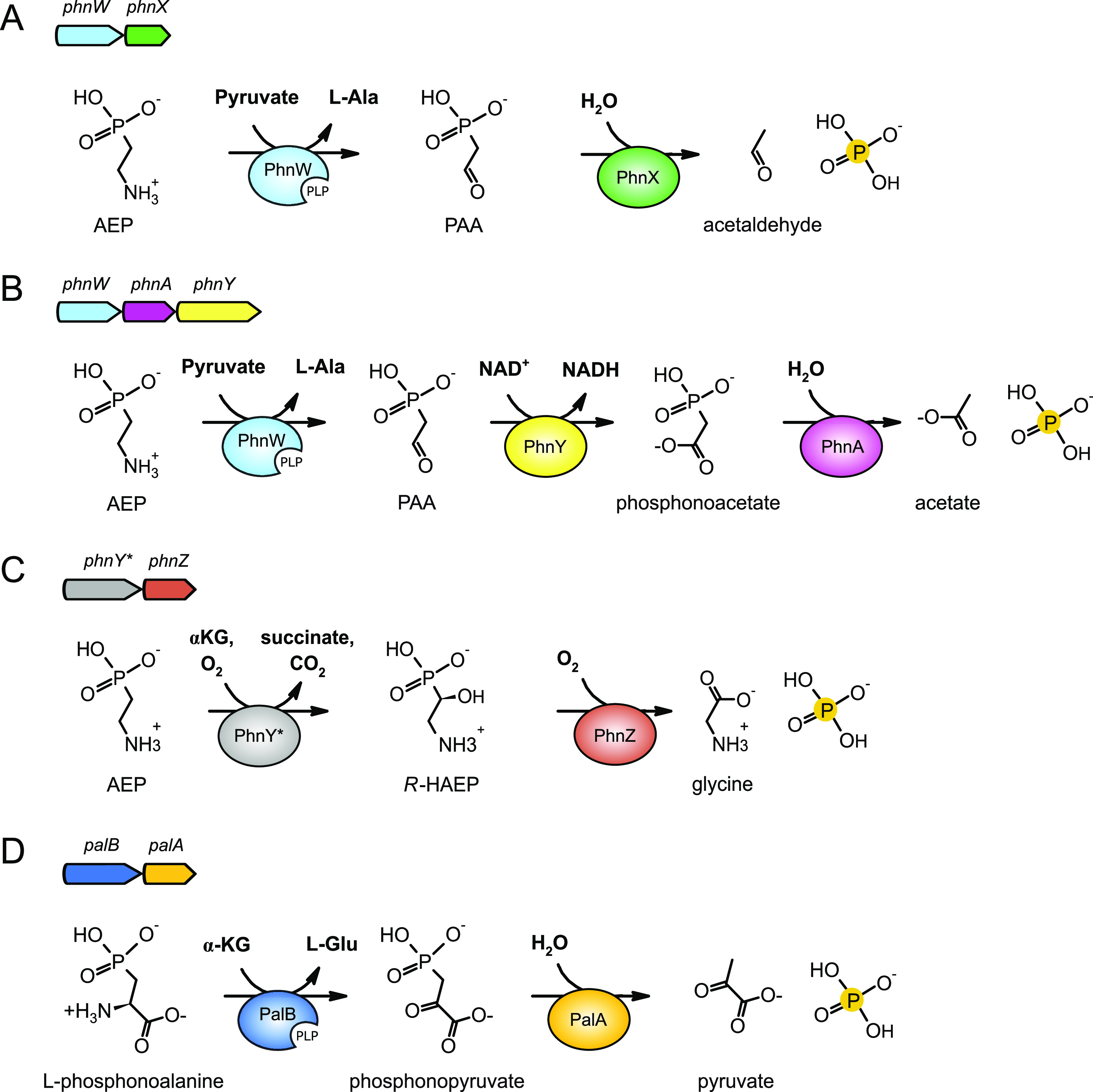
Pathways for the microbial catabolism of aminophosphonates. The organization of the corresponding genes into genomic clusters is schematically shown above each panel. (A) AEP hydrolytic degradation. The transaminase PhnW converts AEP to PAA, which in turn is cleaved into acetaldehyde and phosphate by PAA hydrolase (PhnX). (B) Variant of the pathway described above, in which PAA is converted to phosphonoacetate by PAA dehydrogenase (PhnY) and finally to acetate and phosphate by phosphonoacetate hydrolase (PhnA). (C) AEP oxidative degradation, proceeding through the consecutive reactions of two dioxygenases (PhnY* and PhnZ) and formation of an *R*-HAEP intermediate; the final products are glycine and phosphate. (D) Degradation of l-phosphonoalanine. The amino acid is first transaminated by PalB, using α-ketoglutarate as the amino group acceptor; the product phosphonopyruvate is then cleaved by the hydrolase PalA into pyruvate and phosphate.

As shown in Figure 1, PLP-dependent enzymes play a pivotal role in different routes of aminophosphonate degradation. Because it is certain that additional enzyme systems (beyond those characterized so far) exist for the catabolism of phosphonates, we performed a detailed analysis of the genomes of many microorganisms that degrade phosphonates, looking in particular for genes that encode PLP-dependent enzymes and are associated with gene clusters for aminophosphonate breakdown.

This analysis led us to one gene that is often found within clusters containing the *phnW–phnX* or *phnW–phnY–phnA* combination. We termed this gene *pbfA*. After hypothesizing possible functions for the gene product (compatible with the known catalytic range of PLP-dependent enzymes), we recombinantly produced PbfA from the marine bacterium *Vibrio splendidus* and assessed its activity *in vitro* against the potential substrates. Our results demonstrate that PbfA, despite being consistently annotated as a transaminase, is in fact a lyase acting on *R*-HAEP, a natural compound.^14,16,18,19^ The reaction catalyzed by PbfA converts *R*-HAEP into PAA, which can be subsequently processed by PhnX.

## Materials and Methods

### Materials

Alcohol dehydrogenase (ADH, baker’s yeast), glutamate dehydrogenase (GDH, beef liver), and d,l-phosphonoalanine were from Sigma. 2-Aminoethylphosphonate (AEP) was from Wako chemicals. Triethanolamine (TEA), pyridoxal 5′-phosphate (PLP), and bovine serum albumin (BSA) were from Sigma-Aldrich. NADH was from Alfa Aesar. TLC plates (silica gel 60 F_254_) were from Merck Millipore. Deuterium oxide was from VWR International, and deuterated trimethylsilyl propionic acid (TSP) from Stohler Isotope Chemicals. All other reagents were from Fluka or Sigma-Aldrich.

Racemic HAEP was synthesized beginning from vinylphosphonic acid, as described in ref (15); the *S* and *R* enantiomers of HAEP were also prepared by methods described in the same publication.^15^

### Bioinformatics Analyses

For the analysis of bacterial genes involved in aminophosphonate breakdown and *pbfA* identification, we performed protein homology searches with the web tools BlastP^20^ and the Integrated Microbial Genomes & Microbiomes (IMG/M) (https://img.jgi.doe.gov/m/). The genomic contexts were visualized and analyzed using tools available on NCBI, IMG/MER, and on the MicrobesOnline Web site (http://www.microbesonline.org/). The similarity of PbfA to functionally validated PLP-dependent enzymes was assessed by consulting the B6 database.^21^ Multiple-sequence alignments were created with ClustalX2 and displayed with ESPript 3.0.^22^

To obtain a census of aminophosphonate degradative gene clusters and assess the frequency of *pbfA*, we downloaded, at the end of November 2020, the protein FASTA assemblies (.faa) of 19,425 complete bacterial genomes deposited in the NCBI Assembly database [https://www.ncbi.nlm.nih.gov/assembly/?term=(Bacteria%5Borgn%5D+OR+Archaea%5Borgn%5D)+AND+(latest_refseq%5Bfilter%5D] using the following parameters: Bacteria[orgn] AND (latest_refseq[filter]) AND (latest[filter] OR “latest refseq”[filter]) AND “complete genome”[filter] AND “full genome representation”[filter] AND all[filter] NOT anomalous[filter].

All of the other steps were automated through a python3 script. The downloaded proteomes were indexed as a database to perform a local homology search with DIAMOND BLASTP^23^ by setting an *E*-value threshold of 1 × 10^–5^ and using as queries the sequences of the following functionally characterized enzymes: PbfA from *V. splendidus* (NCBI reference sequence WP_004730150.1), PhnW from *Salmonella enterica* (NCBI reference sequence NP_459426.1), PhnX from *S. enterica* (NCBI reference sequence NP_459427.1), PalB from *Variovorax* sp. *Pal2* (GenBank entry ABR13824.1), PalA from *Variovorax* (GenBank entry AAO24736.1), PhnY from *Sinorhizobium meliloti* (GenBank entry RMI18174.1), PhnA from *S. meliloti* (GenBank entry AAC15507.1), PhnY* (GenBank entry ACU83549.1), PhnZ (GenBank entry ACU83550.1), and the phosphonopyruvate decarboxylase (Ppd) from *Bacteroides fragilis* (NCBI reference sequence WP_011202932.1). The hits found for each query were tagged with their blast query name (e.g., “pbfA”) and numbered according to their order in the .faa files. The numbered tagged hits of each proteome were scanned with DBSCAN^24^ from the scikit-learn module^25^ to find putative clusters that had at least two hits (min_samples = 2) at no more than eight numbers away (eps = 8). The obtained putative clusters were filtered considering only the known phosphonate catabolism clusters (*phnWX*, *phnWAY*, *palAB*, and phnY*Z), and their corresponding variants that include *pbfA* and/or *phnZ*. Because *palA* is a close homologue of the phosphoenolpyruvate mutase coding gene, which forms with *ppd* the phosphonate biosynthetic gene cluster, we excluded from the results all of the *palAB-ppd* cases.

### Plasmid Constructs and Protein Purification

The *V. splendidus* 12B01 genes for PhnW (NCBI reference sequence WP_004730149.1), PhnX (NCBI reference sequence WP_004730152.1), and PbfA (NCBI reference sequence WP_004730150.1) were codon-optimized for expression in *Escherichia coli*, synthesized, and inserted into pET24a-C-HIS, pET28a-N-HIS, and pBluesScript II KS(+), respectively, by BaseClear BV (Leiden, The Netherlands).

The *pbfA* gene was later subcloned into a pET28-CpoI expression vector^26^ exploiting the CpoI restriction site, to express an N-terminal His6-tagged protein. The *E. coli* XL1-Blue strain was used for cloning applications and plasmid amplification. The subcloned *pbfA* gene was sequence-verified, and the plasmid was used to transform *E. coli* Tuner cells (EMD Biosciences) for protein expression. PhnX was also overexpressed in Tuner cells, whereas PhnW was expressed in *E. coli* BL21 star cells.

Cultures were grown at 37 °C in LB medium supplemented with kanamycin (50 μg/mL) until the OD_600_ reached ∼0.7, at which point the temperature was decreased to 20 °C and protein production was induced by adding 0.3 mM isopropyl β-d-1-thiogalactopyranoside (IPTG). Twenty hours after being induced, the cells were harvested by centrifugation, and pellets were resuspended in an appropriate lysis buffer [PhnW and PbfA, 50 mM phosphate buffer (pH 7.5), 150 mM NaCl, 5 mM β-mercaptoethanol, 1 mM PMSF, 1 mM benzamidine, 1 mg/mL lysozyme, and 10 μM PLP; PhnX, 50 mM HEPES (pH 7.5), 200 mM NaCl, 5 mM β-mercaptoethanol, 1 mM PMSF, 1 mM benzamidine, 1 mg/mL lysozyme, and 10 mM MgCl_2_].

Then the cell suspensions were sonicated and centrifuged. Cleared cell lysates were loaded on a His-Select cobalt affinity resin (Sigma-Aldrich), and the recombinant proteins were purified following the manufacturer’s instructions. Protein purity was assessed by sodium dodecyl sulfate–polyacrylamide gel electrophoresis (SDS–PAGE) (Figure S2). Fractions with a purity of >90% were pooled and dialyzed against storage buffer: PhnW, 50 mM sodium phosphate (pH 7.5), 300 mM NaCl, 1 mM DTT, and 5 μM PLP; PhnX, 50 mM HEPES (pH 7.5), 1 mM DTT, and 10 mM MgCl_2_; PbfA, 50 mM HEPES (pH 7.5), 150 mM NaCl, 1 mM DTT, and 5 μM PLP.

After dialysis, 10% (v/v) glycerol was added to the enzyme stocks and their concentration was assessed spectrophotometrically. Each stock was subdivided into aliquots (∼0.5 mL each), which were frozen and stored at −80 °C.

Protein concentrations were determined using the following ε_280_ values, estimated from the amino acid sequence by the ProtParam tool (http://web.expasy.org/protparam/): 48,360 M^–1^ cm^–1^ for PhnW, 32,430 M^–1^ cm^–1^ for PhnX, and 46,870 M^–1^ cm^–1^ for PbfA. The protein yields were 22, 50, and 47 mg/L of culture for PhnW, PhnX, and PbfA, respectively.

### Qualitative Analysis of Enzyme Reactions by Thin Layer Chromatography (TLC)

For the qualitative detection of enzyme activity, PhnW and PbfA were incubated at 37 °C in 50 mM TEA-HCl buffer (pH 8.0), in the presence of substrates at the desired concentrations. At the end of the incubation, 2 μL of the reaction mixture was spotted on a thin layer silica gel plate and developed (1:3:1 acetic acid/1-propanol/distilled water). After chromatographic separation, amino group-containing compounds were visualized by spraying the plates with 0.3% ninhydrin in methanol, drying, and heating for ∼5 min.

### Measurement of Phosphate Release

Phosphate release during the course of enzymatic reactions was assessed by using the BIOMOL Green kit (Enzo Life Sciences), according to the manufacturer’s instructions. The reaction mixture contains 50 mM TEA-HCl buffer (pH 8.0), 1 mM DTT, 5 μM PLP, 100 mM KCl, 5 mM MgCl_2_, 2 μM PhnW or PbfA, 2 μM PhnX, 1 mM AEP or *R*-HAEP, and 1 mM pyruvate (if specified). When the reaction involved PhnW (whose storage buffer contained phosphate), the enzyme was dialyzed extensively against a phosphate-free buffer [30 mM Hepes (pH 7.5), 300 mM NaCl, 1 mM DTT, and 5 μM PLP] before use.

The reactions were carried out at room temperature and stopped after 1 h by adding a 12-fold excess of BIOMOL Green. Color development was read 30 min later, by measuring the OD_620_ on a Cary 50 UV–vis spectrophotometer (Varian).

### Nuclear Magnetic Resonance (NMR) Measurements

^1^H NMR spectra were collected with a JEOL ECZ600R spectrometer in non-spinning mode at 25 °C using the DANTE presat sequence for H_2_O suppression. The reaction mixture contained 50 mM potassium phosphate (pH 8.0), 100 mM KCl, 5 mM MgCl_2_, 5 mM substrate (AEP or *R*-HAEP, and pyruvate if needed), enzymes (0.8 μM PhnW or 0.8 μM PbfA, 1 μM PhnX), and 1 mM deuterated TSP, used as an internal chemical shift reference (δ 0.00 ppm), in 450 μL of H_2_O and 50 μL of D_2_O. NMR spectra were processed and analyzed with MestReNova version 12.0.4 (Mestrelab Research).

### Spectrophotometric Enzyme Assays

The PhnW-catalyzed transamination of AEP (with pyruvate as a co-substrate) was measured through a coupled assay with PhnX and ADH.^10^ Solutions for the coupled assay (150 μL final volume) typically contained 50 mM buffer (TEA-HCl, pH 8.0), 0.5 mg/mL BSA, ∼0.25 mM NADH, 5 mM MgCl_2_, and 5 μM PLP, in addition to the enzymes and substrates. Unless otherwise indicated, the reactions were carried out at 25 °C and started by the addition of 0.13 μM PhnW. The reaction was measured by monitoring the disappearance of NADH at 340 nm on a Cary 400 thermostated spectrophotometer (Varian).

The PbfA-catalyzed elimination of *R*-HAEP was also monitored by coupling this reaction with the reactions of PhnX and ADH, or via a coupled assay with GDH. In both cases, the reaction was followed by monitoring the disappearance of NADH at 340 nm.

The coupled assay with PhnX and ADH was typically conducted in TEA-HCl buffer (pH 8.0). The reaction mixture also contained ∼0.25 mM NADH, 5 mM MgCl_2_, 5 μM PLP, PhnX, and ADH, in addition to variable amounts of PbfA and *R*-HAEP.

The coupled assay with GDH, detecting the release of ammonia, was conducted under the same conditions as described above, except that PhnX and ADH were omitted, while the reaction mixture contained 1 mM α-ketoglutarate and 6.7 units of GDH.

Kinetic data were analyzed by nonlinear least-squares fitting to the appropriate kinetic equation (e.g., the Michaelis–Menten equation) using Sigma Plot (Systat Software Inc.).

### Simultaneous Measurement of l-Alanine and Phosphate Formation upon Reaction of PhnW with *R*-HAEP

The formation of l-alanine during the reaction of PhnW with *R*-HAEP and pyruvate was quantitated at predefined times through a discontinuous assay based on alanine dehydrogenase.^27^ At the same points in time, the amount of released phosphate was also quantitated through the BIOMOL Green assay, as described above.

Briefly, the reaction mixture (total volume of 1350 μL) contained 20 mM Hepes (pH 7.5), 100 mM KCl, 5 mM MgCl_2_, 5 mM *R*-OHAEP, 5 mM pyruvate, and 0.8 μM PhnW, which was added last to start the reaction. Every 10 min after the addition of PhnW, two aliquots were taken from the reaction mixture and rapidly quenched. One aliquot (150 μL) was heated at 100 °C for 5 min, then frozen, and later analyzed with the alanine dehydrogenase assay to establish the amount of formed alanine. The other aliquot (18 μL) was added to 200 μL of BIOMOL Green reagent; the absorption at 620 nm of this sample was measured after 30 min to calculate the amount of phosphate released.

For the alanine assay, 100 μL of the sample to be analyzed was supplemented with 51 μL of freshly prepared hydrazine-tris buffer [40 mM Tris-HCl (pH 10), 1 M hydrazine, and 1.4 mM EDTA].

After 30 min at room temperature, NAD^+^ (final concentration of 0.8 mM) and alanine dehydrogenase (40 milliunits/mL) were also added. The absorption at 340 nm of these samples was measured after 50, 100, and 240 min. The amounts of phosphate and alanine formed were calculated on the basis of comparison with a calibration curve, obtained using known concentrations.

## Results

### Bioinformatic Identification of a Putative Transaminase Involved in Phosphonate Degradation (PbfA)

**Figure 2:**
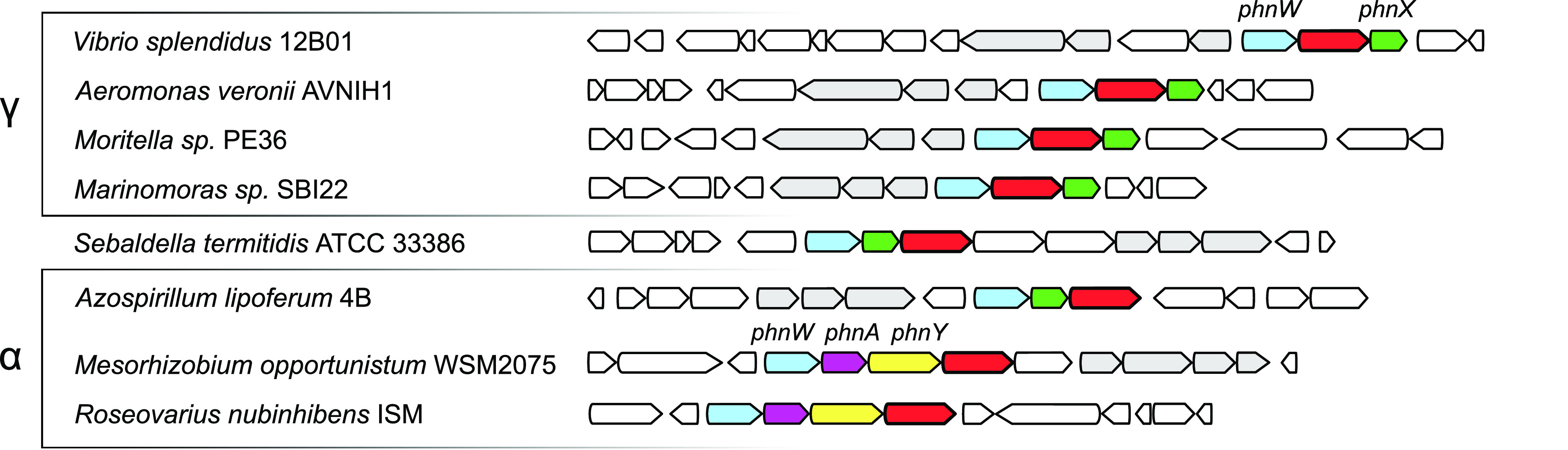
Recurring presence of a putative aminotransferase gene (red) in clusters containing *phnW* (light blue) and *phnX* (green) and in clusters containing *phnW* and *phnA* (purple) and *phnY* (yellow). Phosphonate-related transporter genes are colored gray. Clusters for AEP degradation encompassing the “aminotransferase class III” gene are particularly abundant in γ-proteobacteria and α-proteobacteria but are also found in very distant bacterial lineages, such as *Sebaldella termitidis*, presumably as the result of horizontal gene transfers involving these clusters.^4^

We analyzed the genomic contexts of genes encoding proteins in the C–P hydrolase pathway, beginning with the AEP aminotransferase PhnW. Because it is known that PhnW can play a role both in the biosynthesis and in the degradation of AEP,^12^ we focused on PhnW homologues (>35% identical to the validated enzyme from *Salmonella*(10)) whose genes clustered either with *phnX* or with the *phnA–phnY* duo (*phnWX* and *phnWAY* clusters). On the contrary, we excluded cases in which *phnW* clustered with genes involved in phosphonate biosynthesis (*pepM* and *ppd*). By doing so, we noticed that, in many bacteria, *phnW* and *phnX* were associated with a gene encoding a PLP-dependent enzyme, annotated as 4-aminobutyrate transaminase or, more generically, as a member of the “aminotransferase class III” family (PF00202). This clustering occurred particularly in γ-proteobacteria belonging to the Vibrionales, Aeromonadales, Alteromonadales, and Oceanospirillales orders. Furthermore, in some α-proteobacteria (such as in *Roseovarius nubinhibens*), very similar genes were found associated with the *phnWAY* operon (Figure 2). Due to the recurrent association of this transaminase-like gene with operons dedicated to AEP degradation, we provisionally labeled the encoded protein “phosphonate breakdown factor A” (PbfA).

For homology searches, we used the sequence of PbfA from *V. splendidus* 12B01 (NCBI reference sequence WP_004730150.1) as a query. When BLASTed against the Protein Data Bank (PDB) of structurally characterized proteins, the *Vibrio* sequence showed the greatest similarity to enzymes such as 4-aminobutyrate transaminase from *E. coli* (PDB entry 1SF2, 30% identity) or β-alanine:pyruvate aminotransferase from *Pseudomonas aeruginosa* (PDB entry 4B98, 29% identity). These proteins belong to the Fold-type I structural family of PLP-dependent enzymes,^28^ more specifically to the “aminotransferase class III” subgroup (mentioned above), whose members generally act on substrates that contain an amino group not adjacent to a carboxylate.^29^

A vast majority of functionally characterized “aminotransferase class III” enzymes are indeed aminotransferases, even though the subfamily includes at least one decarboxylase (2,2-dialkylglycine decarboxylase^30^) and a couple of enzymes that catalyze 1,2-eliminations (in particular, ethanolamine-phosphate phospho-lyase^31^).

The similarity of PbfA to β-alanine:pyruvate aminotransferase could be compatible with PbfA catalyzing the transamination of AEP, which is a structural analogue of β-alanine; however, this would be an unneeded duplicate of the reaction catalyzed by PhnW. This argument suggested that PbfA must act on a compound different from AEP and/or catalyze a reaction different from transamination. An alignment of the PbfA sequences with those of other “aminotransferase class III” enzymes showed mutations at some key residues known to be important for substrate and reaction specificity (Figure S1).

Hypotheses about the possible activity of the enzyme were developed on the basis of the following considerations: (a) The substrate of PbfA must necessarily contain a primary amino group, as is the norm with PLP-dependent enzymes. (b) The substrate is presumably a phosphonate compound rather common in nature, to justify the recurrence of the PbfA gene. (c) The product of the reaction should be either AEP or PAA, to feed into the PhnW–PhnX pathway. On the basis of these considerations, two possibilities seemed to be the most convincing. The first was that PbfA could be producing AEP from phosphonoalanine, through a decarboxylation reaction analogous to that catalyzed by dialkylglycine decarboxylase. A second possibility was that PbfA could catalyze a 1,2-elimination on *R-*HAEP (or its enantiomer), to directly generate PAA, a reaction somewhat similar to that of ethanolamine-phosphate phospho-lyase.

Examination of the complete genomes of the bacteria possessing PbfA showed that they almost invariably lacked the known enzymes for l-phosphonoalanine breakdown [PalB and PalA (Figure 1D)] and none of them appeared to possess the only known enzyme for *R-*HAEP degradation [PhnZ (Figure 1C)].

### PbfA Is neither a Transaminase nor a Decarboxylase, but a Lyase Acting on *R*-HAEP

To test the function of PbfA, we recombinantly produced the enzyme from *V. splendidus*, together with PhnW and PhnX from the same organism. We then assessed the activity of purified PbfA *in vitro* against the potential substrates.

First, we tested a possible reaction of PbfA with AEP. When the enzyme was incubated with AEP for at least 1 h, no consumption of the aminophosphonate was observed by TLC. Similarly, when the same experiment was conducted in the presence of an amino group acceptor such as glyoxylate, pyruvate, or α-ketoglutarate, no formation of new amino acids was observed (Figure S3A), implying that PbfA, as predicted, is unable to transaminate AEP. This was confirmed by the fact that no formation of acetaldehyde was detected in a reaction mixture in which PbfA was incubated with AEP and amino group acceptors, PhnX, NADH, and alcohol dehydrogenase (ADH).

Additionally, when PbfA was incubated with d,l-phosphonoalanine under the same conditions as described above, no reaction was detected either by TLC (Figure S3B) or by a coupled assay with PhnW, PhnX, and ADH. These results ruled out the possibility that PbfA could act as a decarboxylase (or as a decarboxylation-dependent transaminase, like dialkylglycine decarboxylase) on phosphonoalanine, to generate AEP or PAA.

**Figure 3:**
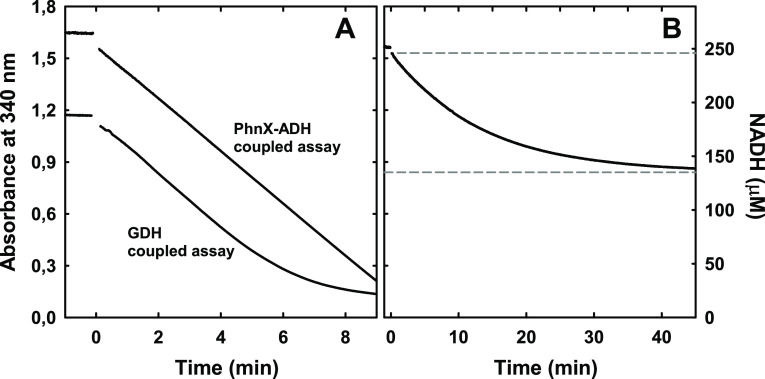
(A) Coupled assays to monitor the reaction of PbfA with HAEP. The assay with GDH monitored the release of ammonia. The reaction mixture contained 10 mM racemic HAEP, 1 mM α-ketoglutarate, ∼0.25 mM NADH, 5 mM MgCl_2_, and 5 μM PLP, in addition to PbfA (2.9 μM) and GDH, in triethanolamine-HCl (pH 8.0). The coupled assay with PhnX and ADH detected the ultimate formation of acetaldehyde. Reaction conditions were as described above, except that α-ketoglutarate and GDH were omitted while PhnX and ADH were included. The very similar slopes obtained in the two assays are consistent with the two processes being both rate-limited by the same step, namely the elimination of water from HAEP. (B) Coupled assay with PhnX and ADH conducted as described above, but in the presence of only 0.2 mM racemic HAEP (and 4.9 μM PbfA). On the basis of NADH consumption, just about 0.1 mM HAEP was used by PbfA, strongly suggesting that only one of the two HAEP enantiomers is a substrate.

In contrast to the results summarized above, we observed that incubation of PbfA with racemic HAEP led to the release of ammonia, as expected in a 1,2-elimination reaction (Figure 3A). The generation of PAA (the other postulated product of the reaction) was inferred by coupling the reaction of PbfA with PhnX and ADH (Figure 3A). Phosphate was not released in the reaction of PbfA with HAEP, except when PhnX was also present [BIOMOL Green assay (data not shown)]. The PbfA-catalyzed reaction consumed only ∼50% of the racemic substrate, implying that only one of the two HAEP enantiomers was used by the enzyme (Figure 3B).

**Figure 4:**
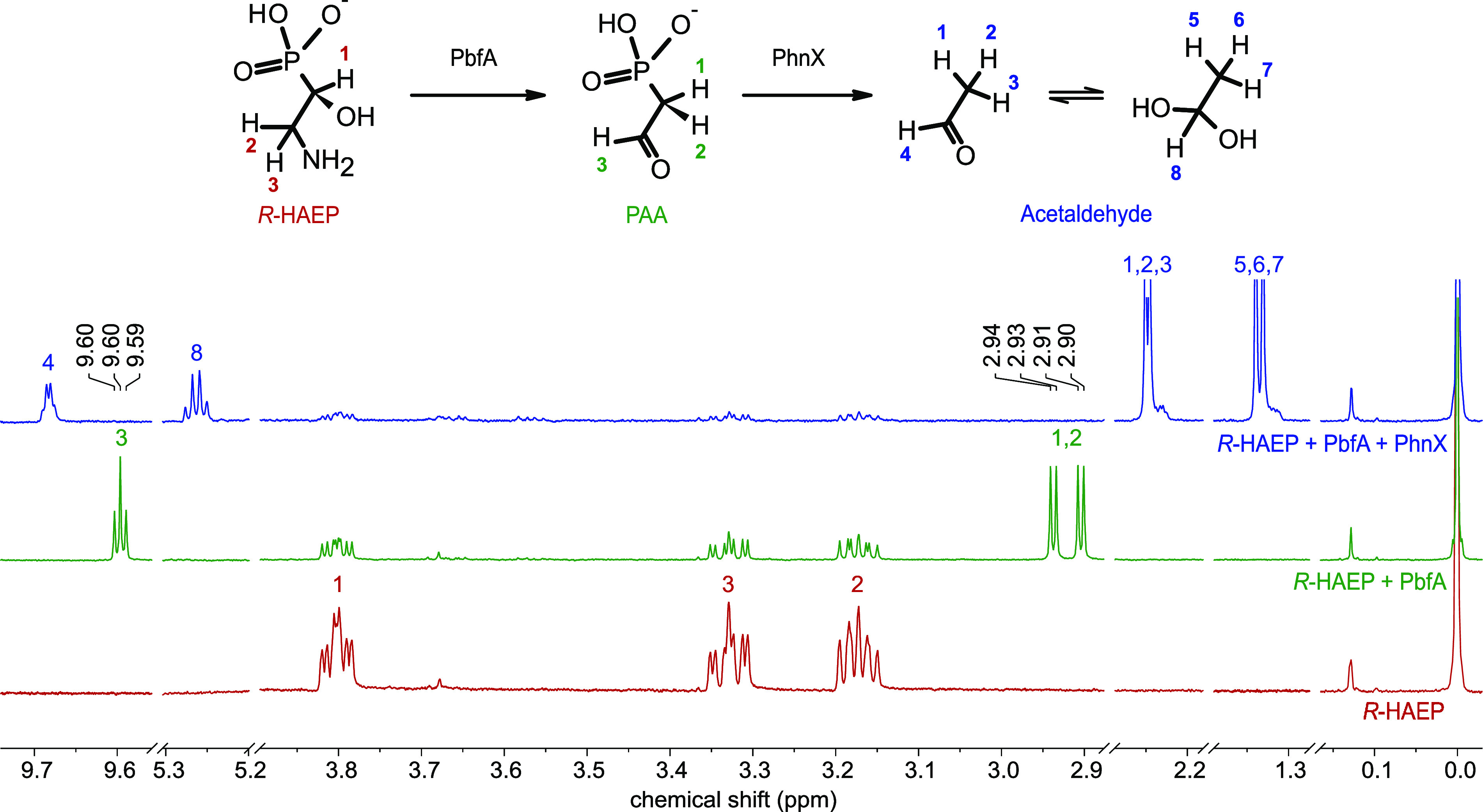
^1^H NMR spectra of *R*-HAEP before (bottom, red) and after (middle, green) a 15 min incubation with PbfA. The new peaks at 2.90–2.94 and 9.60 ppm are attributed to PAA on the basis of published data^32^ and the direct comparison to the spectrum of PAA generated upon transamination of AEP by PhnW (see below). Addition of PhnX (top, blue) led to the disappearance of the peaks mentioned above and to the appearance of new peaks that can be attributed to acetaldehyde. The weak resonances at 3.65 and 3.77 ppm (particularly in the top spectrum) are due to glycerol from the enzymes’ storage buffer. For the attribution of other chemical shifts, see Table S1.

To understand the reaction specificity of the lyase, we synthesized the two HAEP enantiomers as described previously^15^ and tested them individually for reaction with PbfA. These experiments showed that *R-*HAEP is efficiently processed by the enzyme, whereas *S*-HAEP is completely unreactive. When the reaction between PbfA and *R*-HAEP was monitored by ^1^H NMR, consumption of this aminophosphonate was observed, with the simultaneous appearance of a compound showing the ^1^H NMR signature of PAA (t at 9.60 ppm, *J* = 4.25 Hz; dd at 2.90–2.94 ppm, *J* = 19.90 and 4.25 Hz)^32,33^ (Figure 4).

### PbfA Is a Rather Efficient and Highly Specific Enzyme

As shown in Figure 3, we could monitor the kinetics of *R-*HAEP elimination by two different coupled assays. However, the GDH-based assay, despite requiring only one coupling enzyme, was characterized by an initial lag phase that was very hard to eliminate,^34^ presumably due to the complex allosteric behavior of GDH.^35^ Consequently, for the kinetic characterization of PbfA, we used the coupled assay with PhnX and ADH.

**Figure 5:**
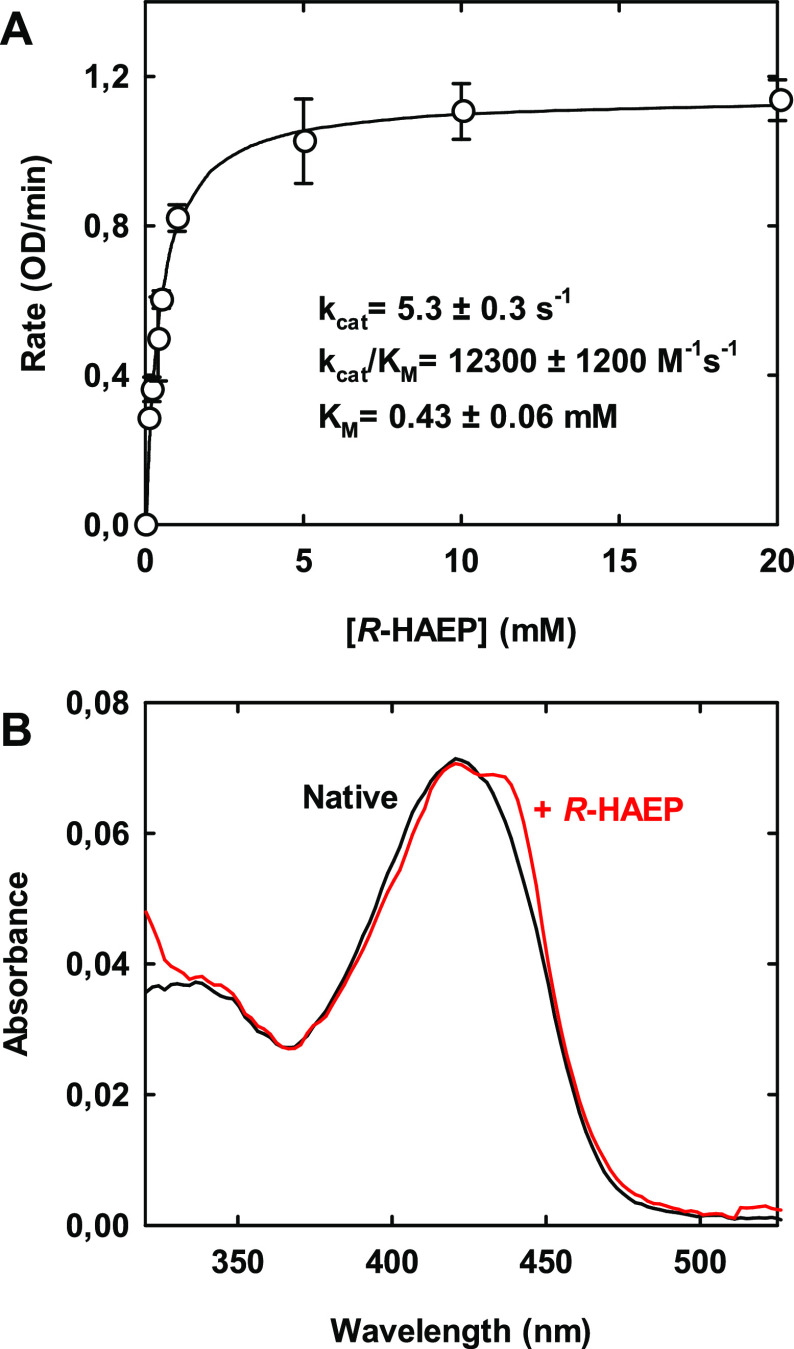
(A) Dependence of PbfA activity on the concentration of *R-*HAEP, as measured by the coupled assay with PhnX and ADH in TEA-HCl buffer (pH 8.0) at 25 °C. The reaction mixture contained ∼0.25 mM NADH, 5 mM MgCl_2_, 0.07% BSA, 5 μM PLP, 0.58 μM PbfA, 4 μM PhnX, and 9 units of ADH. (B) Changes in the ultraviolet–visible spectrum of PbfA (19 μM) before and after the addition of 6 mM *R-*HAEP. Conditions: 50 mM TEA-HCl buffer, pH 8.0, 25 °C, 100 mM KCl, 5 μM PLP, and 1 mM DTT.

At pH 8.0 and 25 °C, the following kinetic parameters were observed: *k*_cat_ = 5.3 ± 0.3 s^–1^, *K*_M_ = 0.43 ± 0.06 mM, and *k*_cat_/*K*_M_ = 12,300 ± 1,200 M^–1^ s^–1^ (Figure 5A). These values are comparable to, or better than, those reported for other PLP-dependent lyases such as ethanolamine-phosphate phospho-lyase.^31,36,37^ They might even be underestimates, because we found that the specific activity of our purified PbfA stocks, once thawed, tended to decrease significantly within a few hours.

Addition of *R-*HAEP to the enzyme caused a small but reproducible red shift of the main absorption band of PLP (Figure 5B), indicative of the formation of some enzyme–substrate adduct, possibly an external aldimine. The spectral change reversed after a few minutes, the time required to completely consume the *R*-HAEP in solution.

Tests on some commercially available *R*-HAEP analogues attested to a strong substrate specificity of PbfA. When the PO_3_ group of HAEP was absent (as in ethanolamine) or replaced by a carboxylate (as in d,l-isoserine, i.e., 3-amino-2-hydroxypropionate), and even if the OH group was substituted by other good leaving groups, such as bromine or a thiol (in bromoethylamine or cysteamine, respectively), the elimination reaction was virtually undetectable (data not shown).

### *R*-HAEP Is Not Efficiently Degraded by PhnW and PhnX

**Figure 6:**
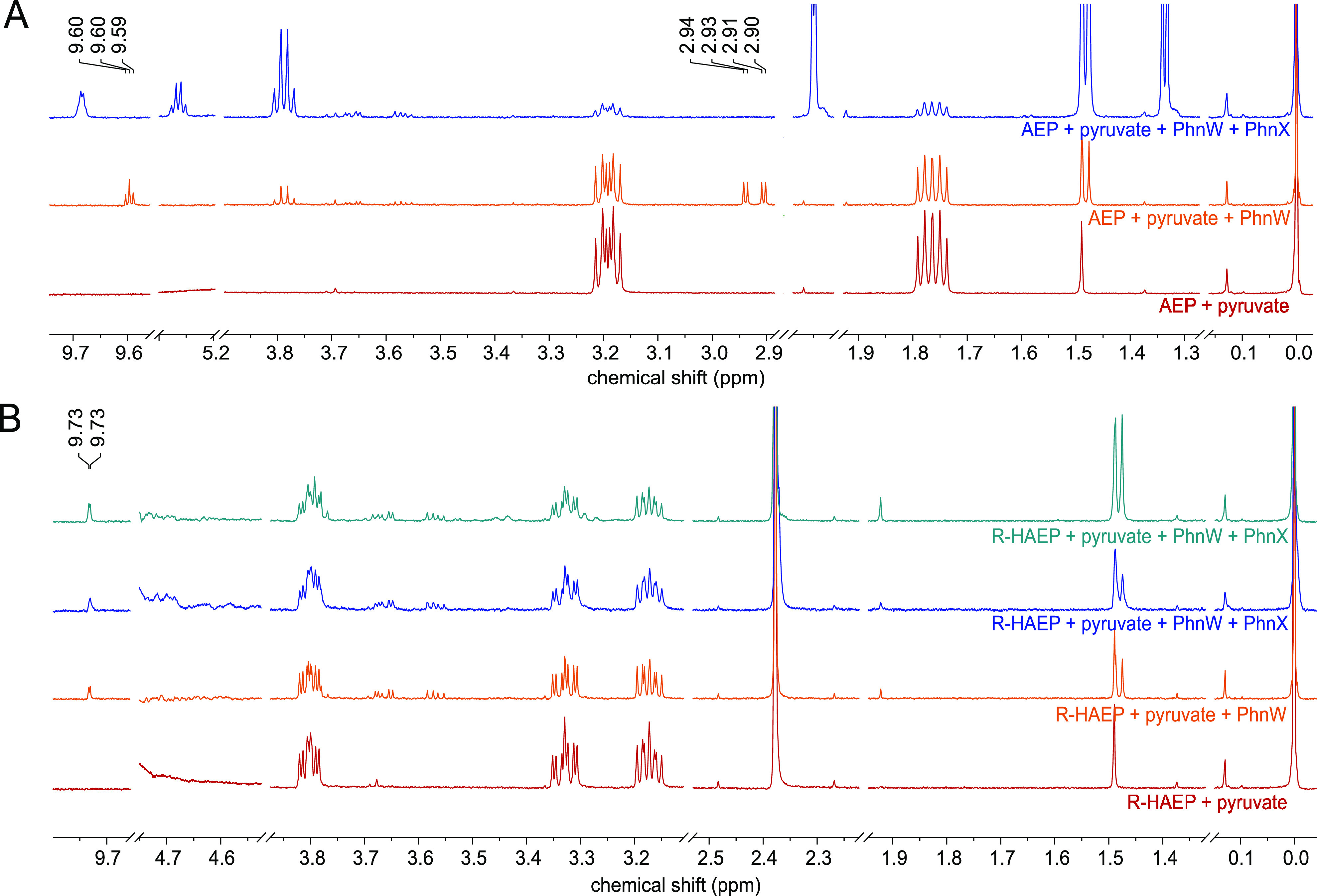
^1^H NMR spectra documenting the reaction of PhnW with AEP and *R*-HAEP. (A) Transamination of AEP. The spectra of a mixture of AEP and pyruvate before (red) and after (orange) a 24 min incubation with PhnW are compared. The new peak at 1.47 ppm corresponds to l-alanine, while the peaks at 2.90–2.94 and 9.6 ppm correspond to PAA. Addition of PhnX (blue) led to disappearance of PAA and to the appearance of new peaks that can be attributed to acetaldehyde. (B) Reaction of PhnW with *R*-HAEP. The bottom spectrum (red) represents a mixture of *R*-HAEP and pyruvate. After incubation with PhnW for >2 h (orange), some new peaks appeared, corresponding to alanine and to an aldehydic proton from an unknown compound [the expected transamination product would be (*R*)-1-hydroxy-phosphonoacetaldehyde]. Addition of PhnX (blue) caused no substantial changes, but some new peaks started to appear after an extended incubation of the mixture (turquoise). The weak resonances at 3.65 and 3.77 ppm can be attributed to glycerol from the enzymes’ storage buffer. For other chemical shifts, see Table S1.

When we incubated the recombinant *V. splendidus* PhnW with its standard substrates, AEP and pyruvate, formation of the expected transamination products, alanine and PAA, was easily detected by ^1^H NMR (Figure 6A). The reaction kinetics could be monitored through the coupled assay with PhnX and ADH, yielding catalytic parameters (*k*_cat_ = 15.5 ± 0.3 s^–1^, *K*_M_^AEP^ = 3.2 ± 0.2 mM, and *k*_cat_/*K*_M_^AEP^ = 4,900 ± 400 M^–1^ s^–1^) comparable to those reported for *S. enterica* PhnW.^10^

When *R-*HAEP was incubated with PhnW and pyruvate, in the absence of PbfA and PhnX, we could observe the formation of alanine by both TLC (Figure S4) and NMR (Figure 6), meaning that *R*-HAEP was being transaminated to some extent. The ^1^H NMR data showed decreases in the intensities of the *R*-HAEP and pyruvate peaks and the appearance of new peaks for alanine, but also for an aldehydic proton (d at 9.73 ppm, *J* = 1.6 Hz), belonging to a compound different from both PAA and acetaldehyde (Figure 6) that did not disappear upon the addition of PhnX.

When we assessed the consecutive reactions of PhnW and PhnX on *R*-HAEP, through a coupled assay with ADH, we did not observe any immediate NADH oxidation, implying that no aldehyde amenable to reduction by ADH was being produced. However, upon incubation of *R*-HAEP with PhnW and pyruvate for 18 h, and finally addition of NADH and a large amount of ADH, we could detect a modest (and rather slow) NADH oxidation, suggesting that some suitable ADH substrate had formed over time. Some small amount of phosphate was also released upon incubation of *R*-HAEP with PhnW, and this release was independent of the presence of PhnX (Figure S5A). In kinetic experiments, at any given time, the amount of phosphate released was much smaller than the amount of alanine formed (Figure S5B), suggesting that phosphate release, albeit dependent on the PhnW transamination reaction, is not tightly coupled to it.

Although we could not positively identify the aldehyde generated by the activity of PhnW on *R*-HAEP, we tentatively interpret the data presented above as follows. We assume that the transamination of *R*-HAEP yields the expected transamination product (*R*)-1-hydroxy-phosphonoacetaldehyde, which however is not a substrate for either PhnX or ADH. With time, 1-hydroxy-phosphonoacetaldehyde breaks down to yield phosphate and glycolaldehyde (which is a substrate of ADH, albeit suboptimal). This “non-enzymatic” breakdown of the C–P bond would be analogous to the reported spontaneous decarboxylation of tartronate semialdehyde, which also yields glycolaldehyde.^38,39^

Irrespective of the actual mechanism, our results show that *R-*HAEP, despite being structurally similar to AEP, cannot be properly processed through the PhnW–PhnX pathway. The importance of a separate processing of *R*-HAEP is also suggested by the fact that homologues of the gene for the oxygenase PhnZ, which converts *R*-HAEP to glycine and phosphate (Figure 1C), are sometimes found to be associated with the *phnWX* or *phnWAY* clusters.^6^ In such cases, PbfA is never present (Table 1).

**Table 1:** Survey of the Occurrence and Composition of Gene Clusters for Aminophosphonate Degradation in Complete Bacterial Genomes

cluster^a^	total	with *pbfA*	with *phnZ*	with *pbfA* and *phnZ*
*phnWX*	1,186	160 (13.49%)	15 (1.27%)	0
*phnWAY*	75	17 (22.7%)	24 (32%)	0
phnY*Z	7	0	–	–
*palAB*	50	0	0	0

a Gene clusters were searched in 19,425 complete bacterial genomes deposited in the NCBI Assembly database as described in Materials and Methods. Genomes that contain *pbfA* (*phnWX-pbfA* and *phnWAY-pbfA*) generally lacked *palAB* and phnY*Z. The only exception was that of *Mesorhizobium terrae* (GCF_008727715.1) that was found to possess both *phnWAY-pbfA* and *palAB* clusters.

## Discussion

### A PLP-Dependent Enzyme with a Novel Lyase Function

The assignment of specific functions to putative enzymes encoded in genomic sequences (particularly if these putative enzymes recur in many species) is a major challenge for biochemistry in the postgenomic era.^40^ The task is hampered by numerous circumstances, for example, when the enzyme possesses an activity never described or proposed before (and hence not predictable on the basis of gaps in metabolic pathways^41^) or when it belongs to a structural family that encompasses a variety of different functions.

The latter situation can be exemplified by considering the so-called Fold-type I structural group of PLP-dependent enzymes. This is the most populated and multifarious among the seven known PLP-dependent “Fold types” (indicated by Roman numerals).^21,28^ Even though all Fold-type I enzymes share both the same cofactor and a conserved overall architecture, they can act on very different substrates and catalyze a striking variety of chemical reactions.^21^ Accordingly, it is difficult to predict *de novo* the activity of these catalysts based on sequence similarity. Furthermore, given their manifold cellular functions, the actual biological role of these enzymes can be difficult to pinpoint even when their catalytic activity has been established.

**Figure 7:**
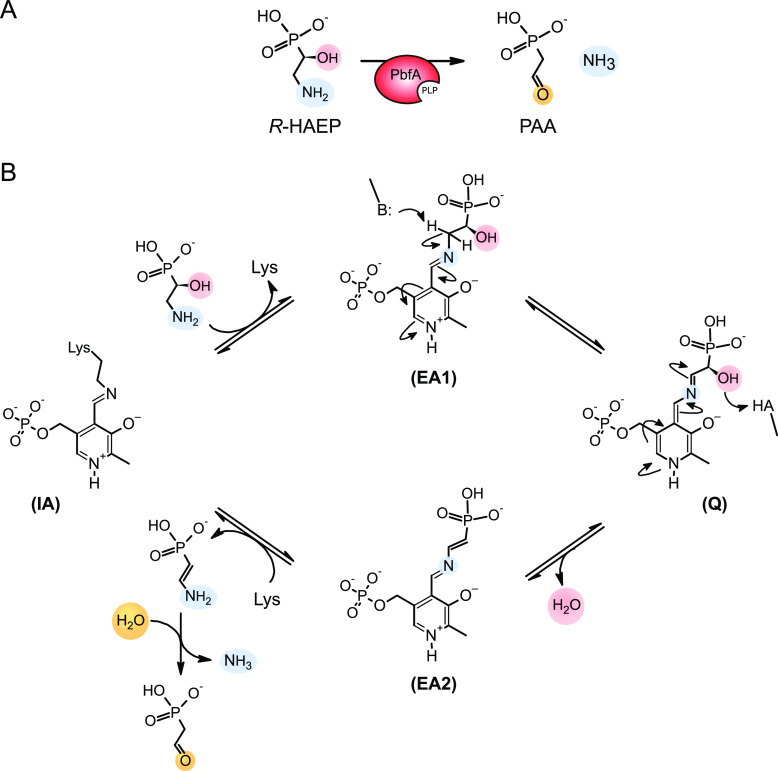
(A) 1,2-Elimination reaction catalyzed by PbfA and (B) proposed mechanism based on that of threonine ammonia-lyase and other PLP-dependent lyases.^42−44^ After entering the enzyme active site, *R-*HAEP can attack the Schiff base formed by PLP with the active site lysine [internal aldimine (IA)] and, passing through a *gem*-diamine intermediate (not shown), complete the transaldimination step to yield the external aldimine (EA1). Abstraction of a proton from the amino carbon, followed by acid-catalyzed elimination of the OH group, can yield the Schiff base of PLP with ethylene 2-amine phosphonate (EA2). These two latter steps may be concerted, in which case a PLP-stabilized carbanion [quinonoid intermediate (Q)] would not form. It is assumed that the eventual hydrolysis of ethylene 2-amine phosphonate (to generate PAA and ammonia) occurs after this compound is released from the active site.^42,43^

Here we report the discovery and functional characterization of a Fold-type I PLP-dependent enzyme, termed PbfA, which is annotated in public databases as 4-aminobutyrate aminotransferase. We have shown instead that PbfA from *Vibrio* (and hence presumably also its close structural and positional homologues found in many bacteria) catalyzes a very specific, and previously undescribed, elimination reaction on (*R*)-1-hydroxy-2-aminoethylphosphonate (*R*-HAEP). This 1,2-elimination reaction is similar to those catalyzed by other PLP-dependent enzymes such as threonine dehydratase (l-threonine ammonia-lyase, EC 4.3.1.19). In analogy with the catalytic mechanism previously put forward for l-threonine dehydratase^42^ and other PLP-dependent lyases,^43,44^ a tentative mechanism for the elimination reaction catalyzed by PbfA can be outlined (Figure 7).

It should be noted that l-threonine dehydratase is not a Fold-type I protein. Indeed, while Fold type I is the most populated structural group of PLP-dependent enzymes,^21^ it encompasses relatively few lyases. This is even more true if one considers the “aminotransferase class III” subgroup, in which the only documented enzymes of this type are ethanolamine-phosphate phospho-lyase and a closely related enzyme,^31^ whose reactions initially release phosphate, rather than water.

PbfA is very substrate-specific, as it shows no appreciable activity toward HAEP-related compounds such as ethanolamine and isoserine, which in principle might undergo the same type of elimination reaction. This observation, together with the constant localization of *pbfA* in gene clusters for aminophosphonate breakdown, implies that the physiological role of this enzyme is strictly dedicated to *R*-HAEP degradation. We hence propose for PbfA the systematic name 1-hydroxy-2-aminoethylphosphonate ammonia-lyase.

### Increasing the Versatility of a Common Phosphonate Degradation Pathway

As noted in the introduction, phosphonates represent an alternative source of P for many microorganisms. This is particularly relevant in marine bacteria, because the marine environment is often poor in bioavailable phosphorus. At least three phosphonate-degrading systems (C–P hydrolases, C–P oxidases, and C–P lyases) have already been identified and characterized at the molecular level. Compared to the C–P-lyase system, which acts on many substrates but requires a multiprotein complex composed by five or more distinct subunit types,^45^ the pathways relying on hydrolases and oxidases appear to be simpler but also restricted to a narrower range of phosphonate substrates.

AEP is the most widely distributed biogenic phosphonate in the environment,^8,12^ so it is not surprising that the specific pathways for the utilization of AEP are present in a variety of bacteria, especially planktonic marine bacteria, including representatives of Proteobacteria, Planctomycetes, and Cyanobacteria. In particular, in 2012, Villarreal-Chiu et al. reported that the PhnW–PhnX pathway was the most abundant phosphonate degradation pathway among 1384 bacteria whose genomic sequences they examined.^4^ Upon analyzing a much larger set of sequences, we found the *phnWX* cluster in 1186 bacterial genomes and showed that in 13.49% of the cases this cluster was enriched by the presence of *pbfA* (Table 1).

The recurrence of *pbfA* in gene clusters dedicated to AEP degradation had been incidentally noted before,^4,6^ but without any experimental follow-up. Our results indicate that PbfA represents a new branch in the well-established hydrolytic AEP degradation pathway and serves to catabolize *R-*HAEP, funneling it into the PhnW–PhnX pipeline. We have shown that, even though HAEP is structurally very similar to AEP, it cannot be properly processed by the PhnW–PhnX pathway, an observation that might justify the very existence of a dedicated degradative enzyme. An additional reason for degrading *R*-HAEP could be its potential toxicity, as it has been reported that racemic HAEP, when taken up by *E. coli*, inhibits growth.^46^ Cells possessing the machinery for importing AEP may find it difficult to completely exclude HAEP; therefore, possessing an enzyme for its breakdown might be advantageous.

The information about the natural abundance of *R*-HAEP is limited. This compound was first isolated as a hydrolysis product of complex polysaccharides of the soil amoeba *Acanthamoeba castellanii*.^18,19^*R*-HAEP is also an intermediate in the oxidative pathway for the degradation of AEP, which relies on the reactions of the oxygenases PhnY* and PhnZ^14,16^ (Figure 1C). Furthermore, the incorporation of HAEP (stereochemistry unspecified) in membrane phospholipids was reported for the predatory bacterium *Bdellovibrio stolpii*(47) and postulated (on genomic grounds) for other bacteria.^48,49^ Despite the scantiness of these data, the very frequency with which *phnWX-pbfA* (or the nearly equivalent combination *phnWAY-pbfA*) occurs among bacteria (Figure 2 and Table 1) suggests that *R-*HAEP may be a rather abundant phosphonate in certain environments.

The ability to consume *R-*HAEP, in addition to AEP, allows a bacterium to exploit different phosphorus sources and hence, presumably, increases its fitness under variable nutrient availabilities. Achieving this result by adding a branch to a catabolic pathway is common, as in the case of the “tributary pathways” through which different hexoses are fed into glycolysis.^50^ Overall, the function of the *phnWX-pbfA* clusters is analogous to that of the phnY*Z clusters, in the sense that either pathway can extract phosphate from both AEP and *R-*HAEP.^14,16^ However, the PhnY* and PhnZ reactions, requiring molecular oxygen, are not going to be functional under anaerobic conditions, whereas the *phnWX-pbfA* cluster is found also in some strict anaerobes such as *S. termitidis* (Figure 2) or *Clostridium butyricum*.

## Conclusions

Many studies have highlighted the role of phosphonate degradation as a tool bacteria employ for surviving in nutrient-limited ecological niches. It has been shown that even some human-made phosphonates, which have been introduced recently and often cause environmental pollution, can be exploited as phosphorus sources by some bacteria.^51,52^ These studies have characterized several pathways for phosphonate breakdown and implied that additional ones must exist. Indeed, the search for such new pathways is actively pursued in the field, one of the major hurdles being the accessibility of biogenic phosphonates, which are often commercially unavailable or (when available) very expensive and not enantiomerically pure.^53^ While the work presented here does not describe a completely new route for phosphonate breakdown, it does describe a novel enzyme, apparently “invented” by evolution to increase the utility of the common (but otherwise very specialized) hydrolytic pathway for AEP degradation.

## Supporting Information Available

^1^H NMR resonances for a number of compounds studied in this work (Table S1), sequence comparison between PbfA and other phylogenetically related PLP-dependent enzymes (Figure S1), an SDS–PAGE gel of the purified *V. splendidus* enzymes used in this study (Figure S2), TLC plates documenting the lack of activity of PbfA toward AEP and phosphonoalanine (Figure S3), a TLC plate documenting the reactivity of PhnW toward *R*-HAEP (Figure S4), and data for the reaction of PhnW with *R*-HAEP (Figure S5) (PDF)

## Accession Codes

*V. splendidus* 12B01 enzymes (characterized experimentally): PbfA, WP_004730150.1; PhnW, WP_004730149.1; PhnX, WP_004730152.1. Protein sequences mentioned in the bioinformatic analyses: PbfA_S.ter, WP_012862841.1; PbfA_M.opp, WP_013896952.1; PbfA_A.lip, WP_014188790.1; GABA-T_E. coli, NP_417148.1; BA-PAT_P.put, P28269.1; DGD_B.cep, P16932.3; ETNPPL_H.sap, NP_112569.2; PhnW_S.ent, NP_459426.1; PhnX_S.ent, NP_459427.1; PalB, ABR13824.1; PalA, AAO24736.1; PhnY, RMI18174.1; PhnA, AAC15507.1; PhnY*, ACU83549.1; PhnZ, ACU83550.1; Ppd, WP_011202932.1.

## Abbreviations

AEP: 2-aminoethylphosphonate
HAEP: 1-hydroxy-2-aminoethylphosphonate
PAA: phosphonoacetaldehyde
PhnW: 2-aminoethylphosphonate:pyruvate aminotransferase
PhnX: phosphonoacetaldehyde hydrolase (phosphonatase)
PhnY: phosphonoacetaldehyde dehydrogenase
PhnY*: 2-aminoethylphosphonate dioxygenase
PhnZ: 1-hydroxy-2-aminoethylphosphonate dioxygenase
PhnA: phosphonoacetate hydrolase
PalA: phosphonopyruvate hydrolase
PalB: phosphonoalanine aminotransferase
ADH: alcohol dehydrogenase
GDH: glutamate dehydrogenase
PLP: pyridoxal 5′-phosphate
TEA: triethanolamine.
