# Supplementary material for: Discovery of a New, Recurrent Enzyme in Bacterial Phosphonate Degradation: (R)-1-Hydroxy-2-aminoethylphosphonate Ammonia-lyase
Source: Biochemistry. 2021 Apr 8;60(15):1214–25. doi: 10.1021/acs.biochem.1c00092 (PMC8154272; doi:10.1021/acs.biochem.1c00092)
Supplement: Supplementary file 1 — bi1c00092_si_001.pdf [file bi1c00092_si_001.pdf]

SUPPORTING INFORMATION FOR:

Discovery of a new, recurrent enzyme in bacterial phosphonate degradation: (*R*)-1-hydroxy-2-aminoethylphosphonate ammonia-lyase

*Erika Zangelmi[a], Toda Stanković[b], Marco Malatesta[a], Domenico Acquotti[c], Katharina Pallitsch[b], Alessio Peracchi[a]**

[a] Department of Chemistry, Life Sciences and Environmental Sustainability, University of Parma, I-43124 Parma, Italy.

[b] Institute of Organic Chemistry, University of Vienna, Währingerstrasse 38, A-1090 Vienna, Austria.

[c] Centro di Servizi e Misure "Giuseppe Casnati", University of Parma, I-43124 Italy.

* Corresponding author. Email: alessio.peracchi@unipr.it

SUPPLEMENTARY TABLES

Table S1 – ^{1}H chemical shifts and multiplicity of the compounds studied in this work.

**Compound**	**Structure**	**Proton number**	**δ ^{1}H (ppm) and multiplicity**
2-aminoethylphosphonate (AEP)	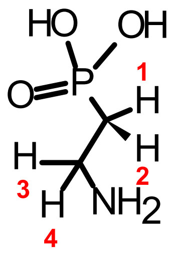	1, 2<br>3, 4	1.74–1.79 (dt)<br>3.17–3.21 (dt)
Phosphonoacetaldehyde (PAA)	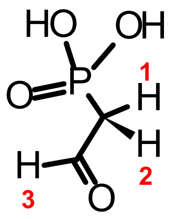	1, 2<br>3	2.90–2.94 (dd)<br>9.59–9.60 (t)
1-hydroxy-2-aminoethylphosphonate (HAEP)	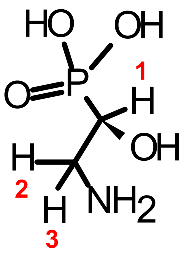	1<br>2<br>3	3.15–3.20 (m)<br>3.31–3.35 (m)<br>3.78–3.82 (m)
Pyruvate hydrate	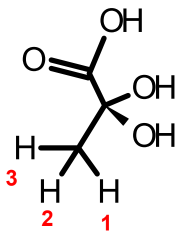	1, 2, 3	1.49 (s)

L-alanine	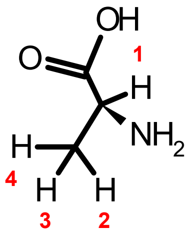	1<br>2, 3, 4	3.77–3.80 (q)<br>1.47–1.49 (d)
Acetaldehyde	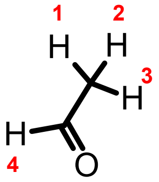	1, 2, 3<br>4	2.25 (dd)<br>9.68–9.69 (d)
Acetaldehyde hydrate	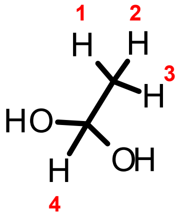	1, 2, 3<br>4	1.33–1.34 (dd)<br>5.25–5.28 (m)
Glycerol	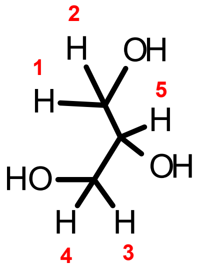	1, 3<br>2, 4<br>5	3.65–3.67 (dd)<br>3.55–3.58 (dd)<br>3.77 (m)

SUPPLEMENTARY FIGURES

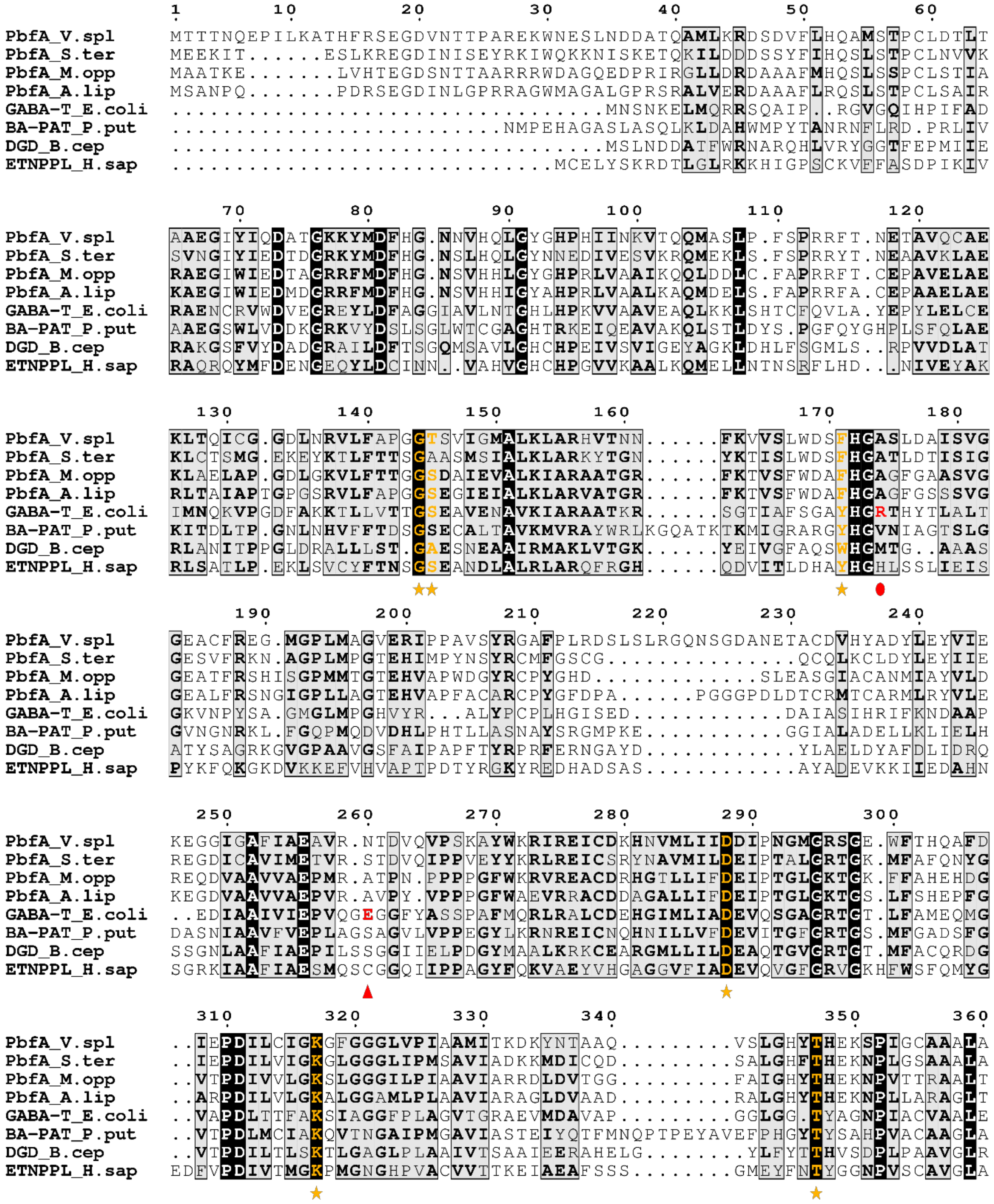

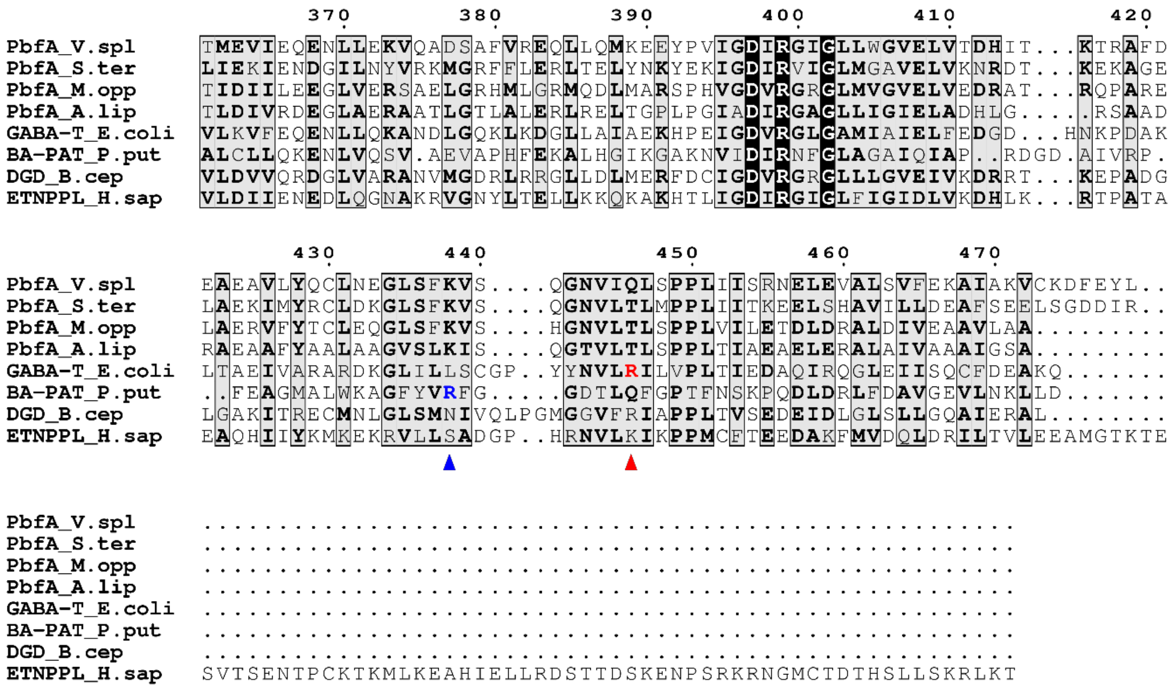

Figure S1 – Multiple sequence alignment of four PbfA proteins (corresponding to genes signaled as putative aminotransferases in Figure 2 of the main text) with four functionally validated enzymes within the subgroup of so-called 'class III aminotransferases', namely, 4-aminobutyrate transaminase from *E. coli* (GABA-T_E.coli; NP_417148.1), dialkylglycine decarboxylase from *Burkholderia cepacia* (DGD_B.cep; P16932.3), ethanolamine-phosphate phospho-lyase from *Homo sapiens* (ETNPPL_H.sap; NP_112569.2) and β-alanine:pyruvate transaminase from *Pseudomonas putida* (BA-PAT_P.put; P28269.1). The orange stars indicate residues that interact with the PLP cofactor and are evidently conserved in PbfA. These include (taking as a reference the sequence of PbfA from *Vibrio splendidus* - PbfA_V.spl) T347, G144 and T145, which bind the phosphate group; D288, which interacts with the pyridine nitrogen; F171, which stacks onto the PLP ring; and the catalytic Lys residue, K317. Red symbols point to the residues typical of aminotransferases that use α-ketoglutarate (α-KG) as the amino group acceptor: the red triangles indicate the Glu and the Arg of the so-called 'gateway system' (E211 and R398 in GABA-T_E.coli; E211 interacts with R398 in the first half-reaction, preventing transamination of α-amino groups instead ω-amino groups, but the interaction is broken in the second half of the reaction, allowing the correct positioning of the keto acid α-carboxylate [1]) while the red circle indicates the Arg residue (R141 in GABA-T_E.coli) that forms a salt bridge with the γ-carboxylic group of α-KG. In case the substrate lacks a distal carboxylic group, this Arg is replaced by other residues; for example, it is replaced by a His in the lyase ETNPPL and by a Met in DGD. PbfA lacks the two arginines and glutamate typical of AT-II transaminases

using the α-KG as the amino group acceptor. In particular, R141 of GABA-T_E.coli is consistently replaced by an alanine (A174 in PbfA_V.spl). In a sense, PbfA is more similar to β-alanine:pyruvate transaminase, which contains a hydrophobic amino acid (V155 in BA-PAT_P.put) at this position. PbfA sequences also contain a conserved lysine (K438 in PbfA_V.slp) at the same position where we find the arginine in β-alanine:pyruvate transaminase and in other AT-II transaminases that use pyruvate as an amino group acceptor (position signaled by a blue triangle).

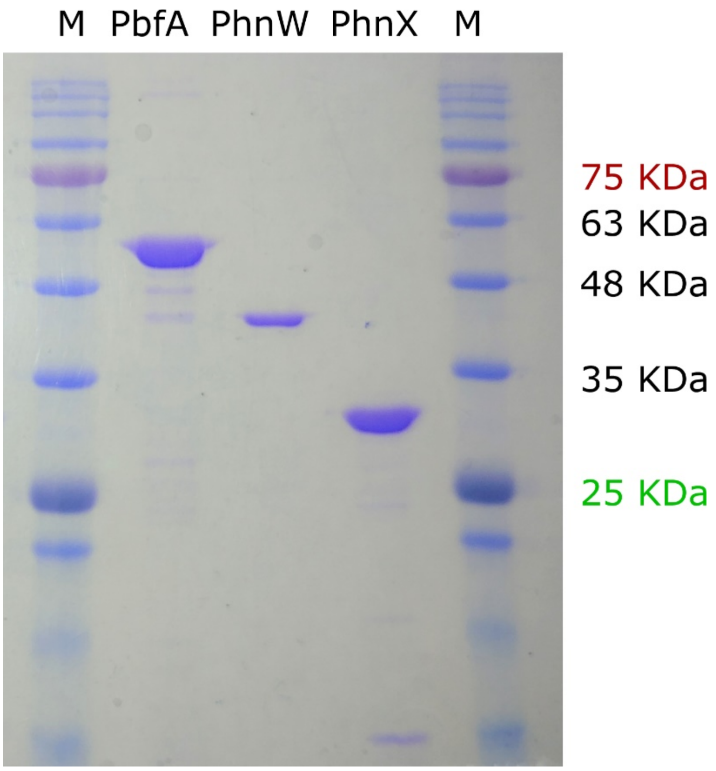

Figure S2 – SDS-PAGE of the purified recombinant proteins used in this study. M: BlueStar Prestained Protein Marker (NIPPON Genetics Europe).

A

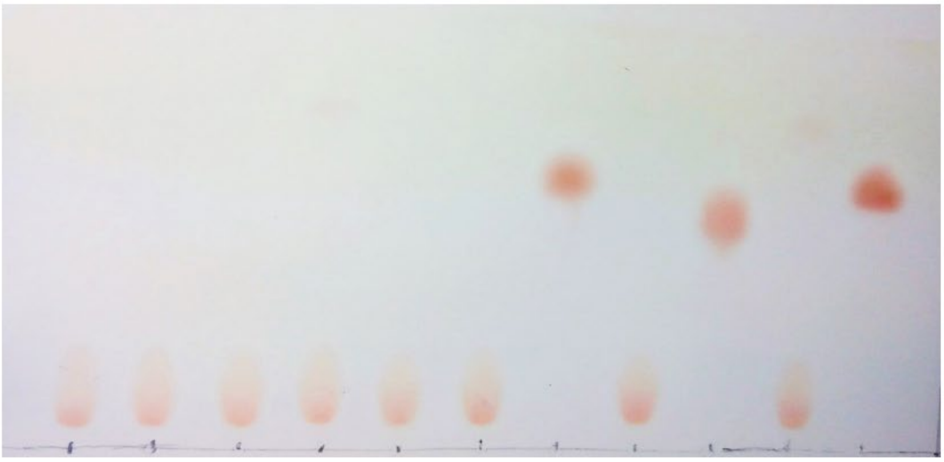

AEP	+	+	+	+	+	+	−	+	−	+	−
Pyr	−	+	−	−	−	+	−	−	−	−	−
Glx	−	−	+	−	−	−	−	+	−	−	−
α-KG	−	−	−	+	−	−	−	−	−	+	−
PbfA	−	−	−	−	+	+	−	+	−	+	−
Ala	−	−	−	−	−	−	+	−	−	−	−
Gly	−	−	−	−	−	−	−	−	+	−	−
Glu	−	−	−	−	−	−	−	−	−	−	+

B

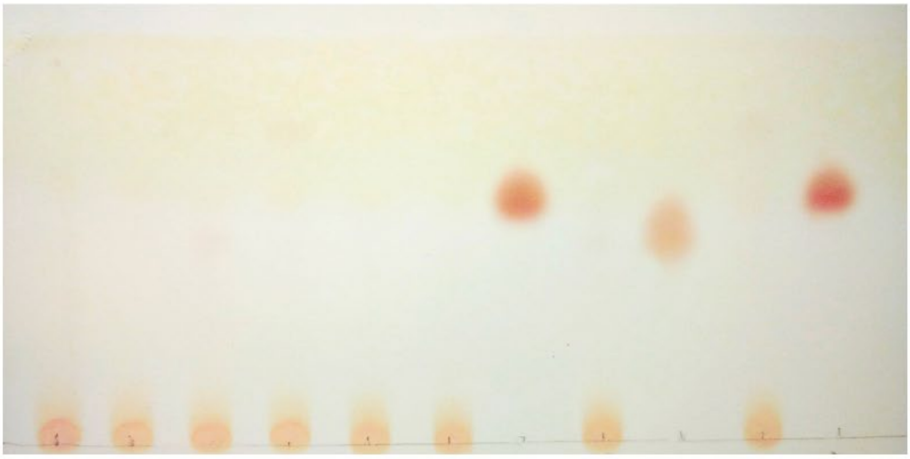

DL-PA	+	+	+	+	+	+	−	+	−	+	−
Pyr	−	+	−	−	−	+	−	−	−	−	−
Glx	−	−	+	−	−	−	−	+	−	−	−
α-KG	−	−	−	+	−	−	−	−	−	+	−
PbfA	−	−	−	−	+	+	−	+	−	+	−
Ala	−	−	−	−	−	−	+	−	−	−	−
Gly	−	−	−	−	−	−	−	−	+	−	−
Glu	−	−	−	−	−	−	−	−	−	−	+

Figure S3 – Lack of activity of PbfA towards AEP and phosphonoalanine. (A) PbfA (1 μM) was incubated for one hour with AEP (10 mM) alone or in the presence of potential amino group acceptors (pyruvate, glyoxylate or α-ketoglutarate; 10 mM each). Conditions: 50 mM TEA-HCl buffer (pH 8.0), 5 mM $MgCl_2$; 150 μl final volume; 37 °C. At the end of the incubation, two microliters of the

reaction mixtures were spotted on a TLC silica gel plate and developed (acetic acid:1-propanol:distilled water - 1:3:1) side by side with controls containing the expected transamination products (L-Alanine, Glycine and L-Glutamate). After chromatographic separation, the amino group-containing compounds were visualized by ninhydrin staining. (B) The possible reaction of PbfA with phosphonoalanine was assayed as above, except that D,L-phosphonoalanine (DL-PA; 10 mM) was used in place of AEP.

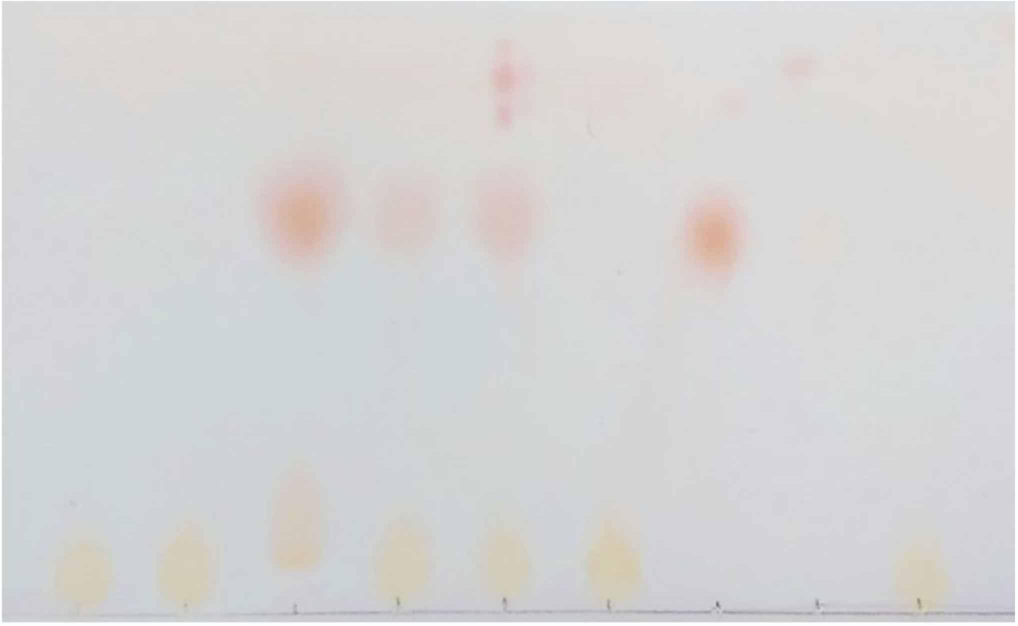

AEP	−	−	+	−	−	−	−	−	−
R-HAEP	+	−	−	+	+	−	−	+	−
S-HAEP	−	+	−	−	+	+	−	−	+
Pyr	−	−	+	+	+	+	−	−	−
PhnW	−	−	+	+	+	+	−	−	−
PbfA	−	−	−	−	−	−	−	+	+
Ala	−	−	−	−	−	−	+	−	−

Figure S4 – Modest reactivity of PhnW towards *R*-HAEP. PhnW (1 µM) was incubated for one hour with 10 mM pyruvate and either AEP (as a control), *R*-HAEP, racemic HAEP or *S*-HAEP (10 mM in each case, except racemic HAEP that was 20 mM). Other conditions: 50 mM TEA-HCl buffer (pH 8.0), 5 mM $MgCl_2$, 5 µM PLP, 1 mM DTT; 150 µl final volume; 37 °C. At the end of the incubation, samples were separated on a TLC plate and visualized as in Figure S3.

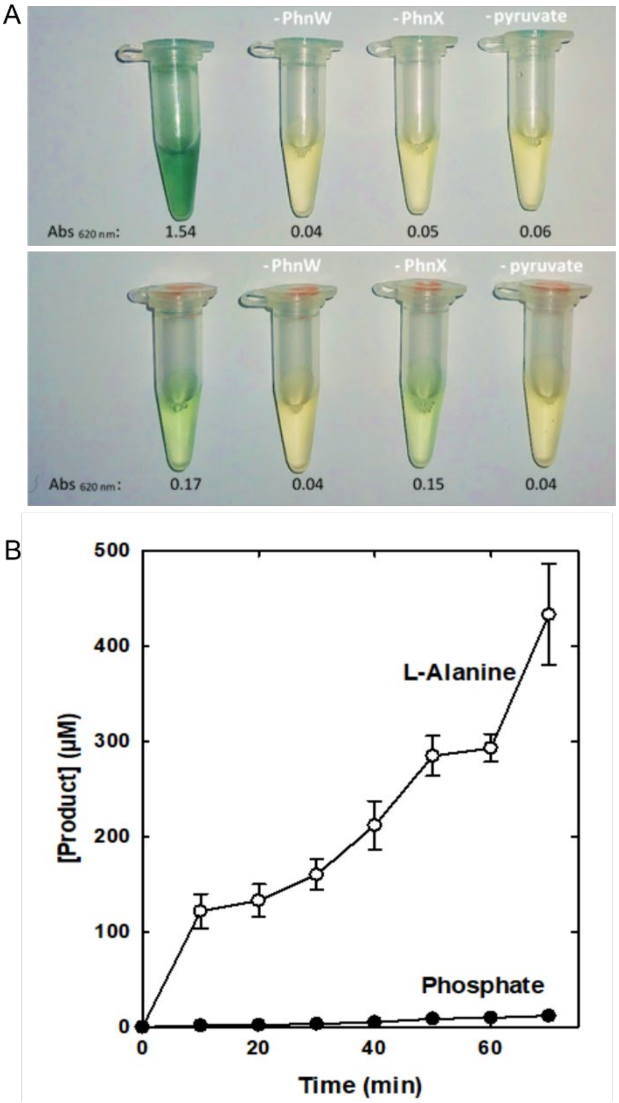

Figure S5 – *R*-HAEP is not efficiently degraded by PhnW and PhnX. (A) Colorimetric detection of phosphate release in the reactions catalyzed by PhnW and PhnY from *V. splendidus* with AEP (upper row) or *R*-HAEP (lower row). In the reaction with AEP, the leftmost tube contained the complete reaction mixture: 50 mM TEA pH 8.0, 1 mM DTT, 5 µM PLP, 100 mM KCl, 5 mM $MgCl_2$, 2 µM PhnW, 2 µM PhnX, 1 mM of AEP and 1 mM of pyruvate. The other three tubes were control reactions in which either PhnW, PhnX or pyruvate were omitted. Reactions were carried out at room temperature and they were stopped by adding the BIOMOL® Green reagent after 1 h. In the reaction with *R*-HAEP (lower row) the experiment was conducted as above, except that *R*-HAEP was used in place of AEP. The greenish color in the first and third tube (but not in the other controls) indicates a weak release of phosphate dependent on the transaminase activity of PhnW, but independent of PhnX. (B) The kinetics of the reaction catalyzed by PhnW with *R*-HAEP indicate that phosphate release is not concomitant with *R*-HAEP transamination. The reaction mixture contained: 20 mM Hepes pH 7.5, 100 mM KCl, 5 mM $MgCl_2$, 5 mM *R*-HAEP, 5 mM

pyruvate and 0.8 μM PhnW. The amounts of L-Alanine and phosphate produced at given time-points were determined through the assays described in the Methods.

SUPPLEMENTAL REFERENCES

1 Schiroli D & Peracchi A (2015) A subfamily of PLP-dependent enzymes specialized in handling terminal amines. Biochim Biophys Acta - Proteins Proteomics 1854, 1200–1211.
